# Development of LactaPedia: A lactation glossary for science and medicine

**DOI:** 10.1111/mcn.12969

**Published:** 2020-02-07

**Authors:** Melinda Boss, Peter Hartmann, Jennifer Turner, Douglas Pritchard, Rafael Pérez‐Escamilla, Rhonda Clifford

**Affiliations:** ^1^ School of Allied Health, Division of Pharmacy The University of Western Australia Perth Western Australia Australia; ^2^ School of Molecular Sciences The University of Western Australia Perth Western Australia Australia; ^3^ School of Medicine, Division of General Practice The University of Western Australia Perth Western Australia Australia; ^4^ Department of Social and Behavioural Sciences Yale School of Public Health New Haven Connecticut

**Keywords:** breastfeeding, humans, infant, information dissemination/methods, lactation, mothers, terminology

## Abstract

During the last decade, there have been several publications highlighting the need for consistent terminology in breastfeeding research. Standard terms and definitions are essential for the comparison and interpretation of scientific studies that, in turn, support evidence‐based education, consistency of health care, and breastfeeding policy. Inconsistent advice is commonly reported by mothers to contribute to early weaning. A standard language is the fundamental starting point required for the provision of consistent advice. LactaPedia (www.lactapedia.com) is a comprehensive lactation glossary of over 500 terms and definitions created during the development of LactaMap (www.lactamap.com), an online lactation care support system. This paper describes the development of LactaPedia, a website that is accessible free of charge to anyone with access to the Internet. Multiple methodological frameworks were incorporated in LactaPedia's development in order to meet the needs of a glossary to support both consistent health care and scientific research. The resulting LactaPedia methodology is a six‐stage process that was developed inductively and includes framework to guide vetting and extension of its content using public feedback via discussion forums. The discussion forums support ongoing usability and refinement of the glossary. The development of LactaPedia provides a fundamental first step towards improving breastfeeding outcomes that are currently well below World Health Organisation recommendations globally.

**Key messages**

- Standard terms and definitions for human lactation are needed for comparison and interpretation of scientific studies and, in turn, to support consistency of healthcare and breastfeeding policy.
- Inconsistent advice is reported by mothers to contribute to early weaning.
- Standard terminology for human lactation is the first step required to support consistent advice.
- This paper describes the development of LactaPedia (www.lactapedia.com), a comprehensive online lactation glossary for science and medicine.

## 1 INTRODUCTION AND BACKGROUND

Most women from high‐income countries now choose to breastfeed, but sustained breastfeeding for the minimum durations recommended by the World Health Organisation (WHO) remains uncommon (Victora et al., 2016). Despite more than 80% of women and their newborns starting breastfeeding, the prevalence of breastfeeding at 12 months of age is lower than 20% in most high‐income countries (Victora et al., 2016).

The significance of the lactating breast is particularly evident when its metabolic activity is considered because the energy output in breastmilk represents about 30% of maternal daily resting energy requirements. This is 5% more than that required by the brain (Boss & Hartmann, 2018a). It is therefore of particular concern that when compared with other major organs such as the heart, brain, liver, lungs, and kidneys, there is a lack of basic research and a lack of translation of that research into the evidence‐based medical care of lactating mothers and their infants (Boss, Gardner, & Hartmann, 2018). To address this, a multidisciplinary team based at The University of Western Australia created LactaMap, a comprehensive online lactation care support system (Boss & Hartmann, 2019). During reviews of the literature required for development of LactaMap, it became clear that many lactation terms had differing definitions. In addition, inconsistent advice from health professionals is a common factor reported by mothers to have contributed to early weaning (Brodribb, 2012; Simmons, 2002). A standard language is the most fundamental starting point towards the provision of consistent advice (Boss & Hartmann, 2018b).

The call for consistent definitions in breastfeeding research is not new (Labbok, Belsey, & Coffin, 1997). The WHO published definitions for six infant feeding categories in an effort to achieve global consensus in 1991 (WHO, 1991). Within the last decade, there have been several publications that have highlighted the need for consistent definitions so that published work can be accurately interpreted, generalisations made (where appropriate) and studies compared (Labbok & Starling, 2012; Mannel, 2011; Noel‐Weiss, Boersma, & Kujawa‐Myles, 2012; Yourkavitch & Chetwynd, 2019). An example of the clinical significance of this absence of standard definitions is highlighted in a systematic review of observational studies to determine the impact of breast reduction surgery on breastfeeding (Kraut et al., 2017). This review concluded that breastfeeding was successful in 100% of women where full preservation of the column of parenchyma from the nipple areola complex to the chest wall was retained. However, the reviewers noted that, “most studies used a definition of breastfeeding success of less than a month.” This definition of success is confusing, not what could be considered normal function and not a reasonable physiological measure of success (Boss et al., 2018). Such a concept of successful breastfeeding, when used by health professionals as advice for women who wish to breastfeed successfully after breast reduction surgery, is likely to encourage surgery prior to the completion of child bearing because it is interpreted to mean that it has no impact on breastfeeding. In fact, consideration should be given to deferring breast reduction plans until after breastfeeding is complete. LactaPedia was conceived as a means of overcoming such problems.

Review of the large body of literature required for LactaMap provided an opportunity to commence the development of a comprehensive glossary of lactation terminology. This would provide the standardisation required to support consistent education and training of health care professionals and their care for both mothers and infants.

A glossary is an alphabetical list of names or phrases (terms) together with text to explain their meanings (definitions) that relate to a particular subject. Navigation of a glossary is supported by a clear structure for the terminology model (Chute (Chute, Cohn, & Campbell, 1998). Frameworks exist that define standards for developing a terminology system or glossary (Chute et al., 1998; Velardi, Poler, & Tomás, 2006), but these do not define strategies suitable for all the principles required for development of a glossary for human lactation. The decision‐making framework described in this paper started by following the ideas of the working group process approach used by the WHO for defining the six infant feeding categories (Labbok & Starling, 2012). The working group process approach involved defining the stages required to achieve the specified goal, in this case, breastfeeding definitions. These stages were then modified and expanded with the addition of principles from other relevant frameworks to create a methodology that met the needs of the lactation glossary.

The aim of this paper is to describe the methodology created for LactaPedia, a lactation glossary for science and medicine that is also a key component of LactaMap.

## 2 METHODS

Consultation with the Human Research Ethics Office at The University of Western Australia confirmed that publication of methodology and process did not require an ethics review.

The methodology created for LactaPedia involved six stages.

Stage 1: Creation of a first draft glossary of lactation terms and definitionsLactation terms and definitions were collected during the literature reviews required for development of LactaMap. All terms relating to the field of human lactation and used in LactaMap were documented and defined. This was initially overseen by the LactaMap working group, which is described in the LactaMap Handbook available in the LactaMap website (Boss & Hartmann, 2019). This allowed the issue of variations in lactation terminology to be discussed and clarified.

**Figure 1:**
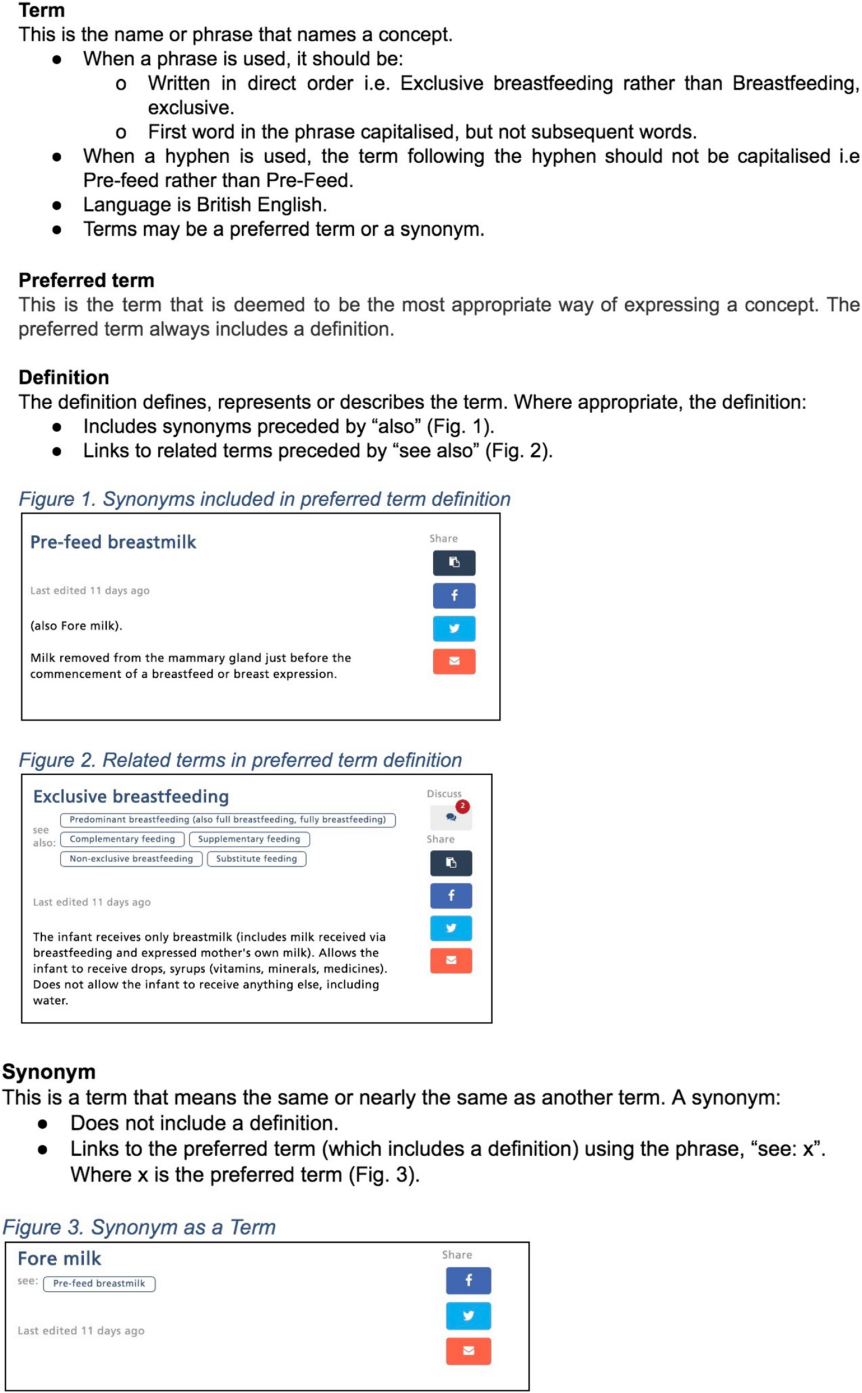
LactaPedia style guide

Using principles from health terminology systems framework, a style guide was created to ensure consistency in lexical rules as well as the attributes of terms, preferred terms, definitions, and synonyms (Figure 1; Chute et al., 1998).

Stage 2: Analysis of first draft glossary and development of inclusion and exclusion criteriaA small, dedicated LactaPedia working group was established to define inclusion and exclusion criteria and reach agreement on terms and definitions to be included in the first draft glossary. The LactaPedia working group consisted of three of the authors (MB, JT, and PH). Additional experts were consulted for input when needed. For example, Dr. Ben Hartmann (author of several papers on the topic of donor human milk and human milk banking) provided input regarding terms relating to donor human milk (Hartmann, 2019; Hartmann, Pang, Keil, Hartmann, & Simmer, 2007). Consensus decision‐making was used to reach agreement within the group. This allowed group members to have collective control over decisions as equals without hierarchy (Seeds for Change, 2010). In keeping with principles of consensus decision‐making that require a clear process and shared understanding on the purpose of the project, a working document called the LactaPedia Handbook was created (Seeds for Change, 2010). The LactaPedia Handbook was developed inductively incorporating principles from existing methodology for terminology standards (Colquhoun et al., 2014; Velardi et al., 2006) as well as those used for evidence‐based guideline development for practical planning and making group decisions (National Institute for health and Care Excellence, 2015; WHO, 2014). The small working group size facilitated open discussion among members. Open discussion is a group decision‐making strategy that makes good use of the knowledge of group members, allowing opinions to be argued (both for and against) and the evidence on which the opinion was based to be assessed (Bang & Frith, 2017).

Once consensus on content was reached, the draft glossary was deemed ready for peer review.

Stage 3: Editorial peer reviewEditorial peer review is a widely accepted element of the scientific process used to assess and improve the quality of publications (Jefferson, (Jefferson, Rudin, Brodney Folse, & Davidoff, 2007). Expert editorial peer reviewers were invited to evaluate the draft glossary developed by consensus of the LactaPedia working group in Stage 2. The approach used for the editorial peer review process was similar to that outlined by The Wiley Network (John Wiley & Sons Inc., 2019): Reviewers were invited to review the draft glossary, the reviews were assessed by the LactaPedia working group, then the draft glossary progressed to Stage 4.

Stage 4: Vetting and refinement based on peer reviewStage 3 produced recommendations regarding acceptance, rejection, or modifications required of the inclusion and exclusion criteria, terms, and definitions. These recommendations provided expertise to guide editorial decisions. The LactaPedia working group retained final decision‐making and further refined the draft glossary based on these recommendations.

Stage 5: Dissemination to a broader audienceThe glossary was disseminated as both a chapter in a print‐based publication and as a website, LactaPedia (https://www.lactapedia.com/lactapedia-site/home) (Boss & Hartmann, 2018; Boss & Hartmann, 2018b). Publication as a website had the dual purpose of facilitating the dissemination and utilisation of the glossary to a broader audience as well as inviting collaborative feedback from users to update and refine LactaPedia.

**Figure 2:**
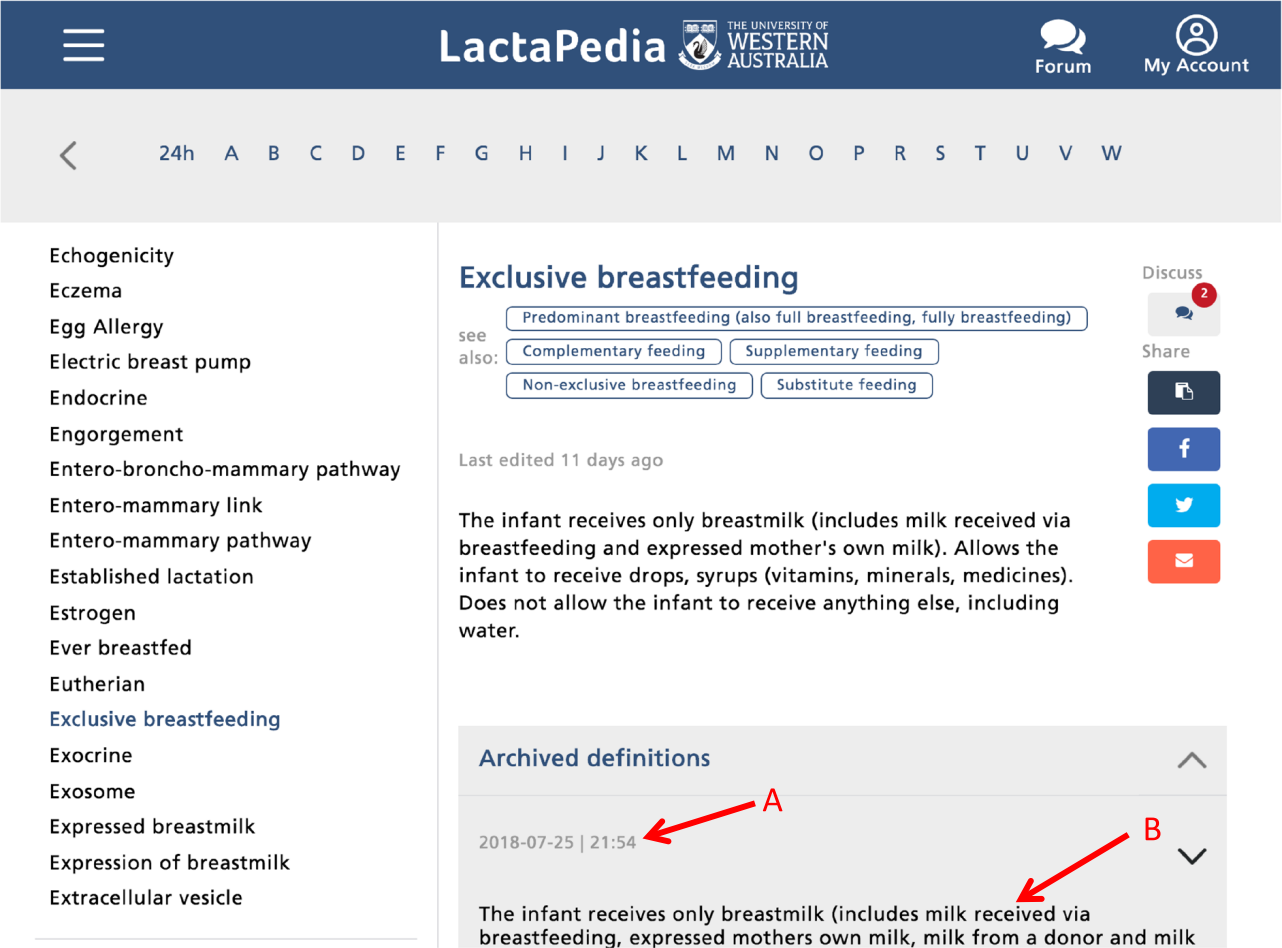
Version control. (a) Date of archiving and (b) archived definition

Version control is a functional characteristic of glossary development important for retaining usability over time (Chute et al., 1998). This allows updates and modifications to be dated and superseded entries to be marked but still be accessible. An innovative aspect of LactaPedia is that entries that become obsolete over time can be retained as archived definitions with the date of archiving recorded (Figure 2).

The online format also allowed particular terms to be highlighted when the user enters the website. The “monthly featured definition” draws attention to a selected specific term and the “newest definition” features the most recent term to be added or amended.

Stage 6: Vetting and extension based on broader public feedbackDevelopment and launch of the LactaPedia website from Stage 5 supported sharing and extension of the glossary by inviting broader public feedback. The final stage involved development of a framework for processing this feedback for update, extension, and refinement of LactaPedia. These principles of update, extension, and refinement are important for ongoing usability and broader acceptance of glossary and terminology systems (Chute et al., 1998; Velardi et al., 2006).

**Figure 3:**
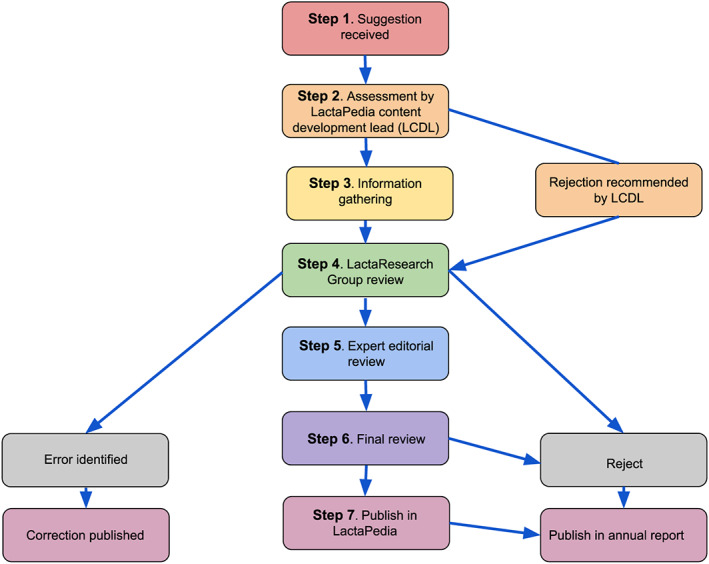
LactaPedia seven‐step review process

Feedback is possible via email, personal communication, and forum comments from the LactaPedia website or directly from the LactaMap working group. Feedback can take one of several forms: a suggested modification of a term or definition, a proposed new term, a proposed new definition requiring a term, and/or identification of an error. All feedback is referred to as a suggestion. At a predetermined date, all suggestions up to but not including that date are recorded in a review process spread sheet. Each suggestion then moves through a seven‐step review process (Figure 3.).

Outcomes for each suggestion are recorded in the review process spread sheet for each of the seven steps. Once complete, the relevant online content can be updated and a report published in the LactaPedia website.

## 3 RESULTS

The methodology developed during the creation of LactaPedia addressed all the requirements considered necessary for a glossary for human lactation.

Multiple methodological frameworks were incorporated in order to meet both the practical needs of consistent health care and decision support as well as the research requirements to facilitate generalisation of findings and comparison of data (Table 1).

**Table 1:** Principles required for LactaPedia and frameworks used

Principle required	Framework used
Effective project planning	Glossary methodology (Velardi et al., 2006)
	Working group process approach (Labbok & Starling, 2012)
	Evidence‐based guideline development methodology (National Institute for Health and Care Excellence, 2015; World Health Organisation, 2014)
Effective decision‐making	Group consensus (Seeds for Change, 2010)
	Open discussion (Bang & Frith, 2017)
Functional characteristics of lexicography	Terminology standards (Chute et al., 1998; Colquhoun et al., 2014)
	Glossary methodology (Velardi et al., 2006)
Objectivity of content	Scientific process (John Wiley & Sons Inc., 2019)
Ability to support update, extension and refinement	Scientific process (John Wiley & Sons Inc., 2019)
	Terminology standards (Chute et al., 1998; Colquhoun et al., 2014)
	Glossary methodology (Velardi et al., 2006)

**Figure 4:**
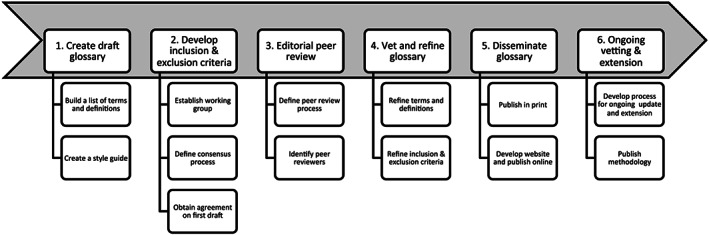
LactaPedia methodology

The resulting LactaPedia methodology was created inductively during the six‐stage process and included principles from all of these frameworks (Figure 4).

LactaPedia launched during World Breastfeeding Week in 2018 and contains more than 560 terms. It is available free of charge to anyone with access to the Internet. Feedback can be submitted by registered users via discussion forums connected to each term or via a general discussion forum accessible from the home page of the website.

## 4 DISCUSSION

LactaPedia is a world first comprehensive human lactation glossary developed using customised methodology.

Lessons learned from LactaPedia were applied when participating in the stakeholder consultation process for the Australian National Breastfeeding Strategy (ANBFS) (Commonwealth of Australia, 2017). An important aspect of the inclusion criteria for LactaPedia is the requirement to define terms in the context of normal function, where possible. For breastfeeding, this required consideration of both the mother and the infant. The aim of the ANBFS is to support the health and well‐being of the next generation by supporting mothers to breastfeed their infants. It seeks to do this by achieving the WHO target for increasing exclusive breastfeeding rates in the first 6 months to at least 50% by 2025 (COAG Health Council, 2019). The original definition of exclusive breastfeeding included in the draft strategy allowed the inclusion of donor human milk and wet nursing. Human lactation is an unusual function in that it optimises health outcomes for a dyad of infant and mother. Including donor human milk and wet nursing in the definition of exclusive breastfeeding preclude the infant's mother from the important health outcomes associated with normal breastfeeding function, increasing the mother's risk for development of some diseases (Ip et al., 2007; Victora et al., 2016). Although donor human milk and wet nursing are helpful aids in the provision of breastmilk to infants, the preservation of a function that also retains the optimal health outcomes of the mother must also be considered when defining a standard to which a national health strategy is aimed. The resulting definition of exclusive breastfeeding included in the published ANBFS reflected this, “exclusive breastfeeding means that the infant receives breast milk (including mother's own expressed breast milk) and allows the infant to receive oral rehydration solution, drops, syrups (vitamins, minerals, and medicines), but nothing else.” (COAG Health Council, 2019).

LactaPedia is currently available in one language (English). The LactaPedia software allows versions in other languages to be included, and work is underway to translate LactaPedia into Chinese. Translation into additional languages is a potential avenue for further research.

The LactaPedia website uses a web analytics service to track website traffic. This provides aggregated statistical information that allows website usage to be tracked. These data do not identify any person individually and may also be used for further research as use of the website progresses.

## 5 CONCLUSION

LactaPedia provides a fundamental first step towards improving breastfeeding outcomes that are less than WHO recommendations globally. The comprehensive, adaptable glossary provides clear, standard terms, and definitions that can be used to support policy as well as generalisation, comparison, and interpretation of research findings and improve consistency of healthcare.

## CONTRIBUTIONS

MB and PH designed and wrote the original study proposal and compiled the first draft of the glossary. MB, PH, and JT developed the methodology. MB significantly contributed to writing this paper. JT, DP, RC, and RPE critically revised the draft manuscript. All authors read and approved the final version of the manuscript.

## CONFLICTS OF INTEREST

The authors declare that they have no conflicts of interest.
